# The tea plant CsLHT1 and CsLHT6 transporters take up amino acids, as a nitrogen source, from the soil of organic tea plantations

**DOI:** 10.1038/s41438-021-00615-x

**Published:** 2021-08-01

**Authors:** Fang Li, Chunxia Dong, Tianyuan Yang, Shilai Bao, Wanping Fang, William J. Lucas, Zhaoliang Zhang

**Affiliations:** 1 State Key Laboratory of Tea Plant Biology and Utilization, Anhui Agricultural University, Hefei, Anhui 230036 China; 2 College of Horticulture, Nanjing Agricultural University, Nanjing, 210095 China; 3 State Key Laboratory of Molecular Developmental Biology, Institute of Genetics and Developmental Biology, Chinese Academy of Sciences, Beijing, 100101 China; 4 Department of Plant Biology, College of Biological Sciences, University of California, Davis, CA 95616 USA

**Keywords:** Plant molecular biology, Plant physiology

## Abstract

Organic tea is more popular than conventional tea that originates from fertilized plants. Amino acids inorganic soils constitute a substantial pool nitrogen (N) available for plants. However, the amino-acid contents in soils of tea plantations and how tea plants take up these amino acids remain largely unknown. In this study, we show that the amino-acid content in the soil of an organic tea plantation is significantly higher than that of a conventional tea plantation. Glutamate, alanine, valine, and leucine were the most abundant amino acids in the soil of this tea plantation. When ^15^N-glutamate was fed to tea plants, it was efficiently absorbed and significantly increased the contents of other amino acids in the roots. We cloned seven *CsLHT* genes encoding amino-acid transporters and found that the expression of *CsLHT1*, *CsLHT2,* and *CsLHT6* in the roots significantly increased upon glutamate feeding. Moreover, the expression of *CsLHT1* or *CsLHT6* in a yeast amino-acid uptake-defective mutant, 22∆10α, enabled growth on media with amino acids constituting the sole N source. Amino-acid uptake assays indicated that CsLHT1 and CsLHT6 are H^+^-dependent high- and low-affinity amino-acid transporters, respectively. We further demonstrated that *CsLHT1* and *CsLHT6* are highly expressed in the roots and are localized to the plasma membrane. Moreover, overexpression of *CsLHT1* and *CsLHT6* in *Arabidopsis* significantly improved the uptake of exogenously supplied ^15^N-glutamate and ^15^N-glutamine. Taken together, our findings are consistent with the involvement of CsLHT1 and CsLHT6 in amino-acid uptake from the soil, which is particularly important for tea plants grown inorganic tea plantations.

## Introduction

Owing to its pleasant flavor and multiple health benefits, tea is the second most popular nonalcoholic beverage worldwide, second only to water. Tea quality largely depends on the contents of polyphenols, caffeine, and theanine in the new shoots used for tea processing. Previous studies have shown that the biosynthesis of these metabolites is related to N (N) conditions^1–3^, with high-quality tea being produced from plants grown under adequate N levels^1,4,5^. However, high N fertilization accelerates soil acidification^6^, which can lead to high accumulations of aluminum, fluorine, and heavy metals (e.g., lead and chromium) in tea leaves, posing potential risks to human health^7^. In recent decades, tea produced from organic plantations has increased in popularity^8,9^. For example, in China, during the past two decades, organic tea production has increased >45-fold^9^.

It has been shown that plants acquire N from the soil in the form of nitrate, ammonium, urea, and amino acids^10^, with amino acids representing a significant N pool in some soils^11–13^. Soil amino acids are derived mainly from exoenzymatic decomposition of proteins and peptides of decaying organisms^14^. Tea plants constitute perennial crops whose leaves are harvested and are periodically pruned 2~3 times per year to maintain vigorous vegetative growth. In this regard, such tea plantation pruning has the potential to produce ~8000 kg ha^−1^ of pruned litter annually^15^. This pruned litter contains high levels of amino acids and proteins that can be recycled following its decomposition in the soil. These pruned litter-derived amino acids may serve as an important N source to be taken up by tea plant root systems.

Plant cells, including those of the roots, take up nutrients through a combination of passive and active transport mechanisms. Channels and permeases can participate in passive uptake when soil nutrient concentrations are high, whereas proton-coupled transporters engage in secondary active transport under low-nutrient conditions^16–18^. Given that soil amino-acid levels are much lower than those within the cells of roots, plasma membrane-localized transporters are generally required for amino-acid uptake from soils^18^. At present, many plant amino-acid transporters have been identified^19–23^ and are grouped into two superfamilies: amino acid/auxin permeases (AAAPs) and amino acid-polyamine-choline transporters (APCs)^16,24^. The AAAP superfamily includes six families, amino-acid permeases (AAPs), lysine and histidine transporters (LHTs), proline transporters (ProTs), GATs (γ-aminobutyric acid transporters), auxin transporters (AUXs), and aromatic and neutral amino-acid transporters (ANTs), whereas the APC superfamily includes members of the cationic amino-acid transporter (CAT) and l-type amino-acid transporter (LAT) families. Amino-acid transporters involved in uptake from the soil belong mainly to the AAP, ProT, and LHT families^25–28^.

In *Arabidopsis*, the LHT family members LHT1 and LHT6 have been shown to be critical for amino-acid uptake by plant roots^25,29^. AtLHT1 displays uptake activity for glutamine (Gln), Ala, Glu, and Asp but not for Arg or Lys. AtLHT6 is involved in the uptake of acidic amino acids (Gln, Ala, and probably phenylalanine) but does not seem to transport basic or other neutral amino acids^25^. Studies devoted to amino-acid transporters have generally been performed on annual plant species, with much less information available on the molecular mechanisms underlying amino-acid transport in perennial species^30–33^.

In this study, we measured the amino-acid composition in the soil at a normal (common) tea plantation and an organic tea plantation. Furthermore, we fed tea plants Glu, the most abundant amino acid detected in the soil and found that it was efficiently absorbed and utilized by these plants. We then cloned seven *CsLHT* genes and determined that CsLHT1 and CsLHT6 were able to transport a broad spectrum of amino acids. *Arabidopsis* lines overexpressing *CsLHT1* and *CsLHT6* were found to exhibit increased uptake of exogenously supplied amino acids. In addition, expression patterns and subcellular localization studies provided support for the hypothesis that CsLHT1 and CsLHT6 play important roles in amino-acid uptake into the roots of tea plants.

## Materials and methods

### Plant culture and amino-acid treatments

Tea plant (*Camellia sinensis* L.) seeds were grown in plastic pots filled with a mixture of soil (40%) and vermiculite (60%). All the pots were irrigated weekly with tap water. The tea plant growth conditions included 16 h of light and 8 h of darkness, 70% relative humidity, and daytime and nighttime temperatures of 25°C and 18°C, respectively. After germination and growth for 100 d, healthy plants with similar crown sizes and heights were selected for studies performed under hydroponic culture. The hydroponic culture method was as described previously by Yang et al.^34^. After 30 d in hydroponic culture, healthy plants with similar crown size and height were selected for amino-acid treatments. Nine seedlings were used for each treatment. For long-term feeding experiments, tea seedlings were transplanted into an N-deficient nutrient solution and allowed to grow for 2 d; afterward, a 1 mM Glu solution was added, and the seedlings were allowed to grow for 5 d. The addition of no Glu served as the control. The tea plant roots were then collected for amino-acid content analysis. For short-term feeding experiments, tea seedlings were transplanted into an N-deficient nutrient solution and allowed to grow for 2 d; afterward, a 2 mM ^15^N-Glu solution was added, and the seedlings were allowed to grow for 6 h or 24 h. The addition of no ^15^N-Glu served as a control. The tea plant roots were then collected for gene expression analysis and ^15^N content determination using a DeltaV isotope ratio mass spectrometer (IRMS; Thermo Fisher Scientific, USA).

Amino-acid feeding to *Arabidopsis* was performed as previously described by Lee et al.^17^. *Arabidopsis* seeds were rinsed in 70% (v/v) ethanol for 1 min followed by sterilization in a solution containing 5% (v/v) NaClO for 15 min. The seeds were then washed four times using sterile water and then vernalized in water for 3 d at 4°C. For amino-acid uptake analysis, the seeds were cultured on 1/2-strength MS solid media for 1 week. The seedlings were then transferred to N-free 1/2-strength MS media that included either 1 mM ^15^N-Glu or 1 mM ^15^N-Gln and allowed to grow for 6 h. These *Arabidopsis* seedlings were then collected to determine the ^15^N content using a DeltaV isotope ratio mass spectrometer. The *Arabidopsis* growth conditions included 16 h of light and 8 h of darkness, 70% relative humidity, and daytime and nighttime temperatures of 21°C and 18°C, respectively.

### Soil sampling and amino-acid extraction and analysis

Amino-acid extraction was performed as previously described by Li et al.^35^. Soil samples were collected from both a control (normal) fertilized tea plantation (Shizipu), located in Xuancheng, and an organic tea plantation (Dayangdian), located in Hefei, Anhui Province, China. The normal (control) tea plantation was fertilized, annually, whereas at the organic tea plantation chemical fertilizer had not been applied during the past 3 consecutive years. Soil samples were collected as follows: the humus in the surface soil was removed, and then soil to a depth of 10 cm was then collected, using a soil sampler; roots and other litter were then removed and 10 g soil samples were taken for drying, with three independent replicates being performed. Subsequently, 10 ml of water was added to each 4 g aliquot of dried soil and then incubated at 70°C for 12 h to extract amino acids. The samples were then cooled to room temperature, followed by centrifugation at 6000 × *g* for 10 min, after which the supernatants were then filtered through a 0.22 μm membrane. The filtrates were subsequently analyzed via an amino acid analyzer (Hitachi, L-8900, Japan).

### Determination of amino-acid contents in tea plant roots

Amino acids were extracted from 50 mg aliquots of freeze-dried tea plant roots using 5 ml of double-distilled water by boiling at 100°C for 20 min. The samples were then cooled to room temperature before centrifugation at 6000 × *g* for 10 min, after which the supernatants were then filtered through a 0.22 μm membrane. The filtrates were analyzed as described above.

### Total RNA extraction and real-time quantitative RT-PCR analysis

Real-time quantitative RT-PCR was performed, as previously described by Li et al.^35^. For tissue-specific expression analysis, total RNA was isolated from buds, stems, vascular bundles, first leaves, leaf veins of the first and third leaves, leaf veins of the third and fifth leaves, leaf veins of the fifth leaf, and roots using an RNAprep Pure Plant Plus Kit (polysaccharides & polyphenolics-rich) (TIANGEN, Beijing, China). The RNA concentration and integrity were evaluated via a Nanodrop 2000 spectrophotometer (Thermo Fisher Scientific, Wilmington, DE, USA) and confirmed via gel electrophoresis. Total RNA (1 μg) was reverse-transcribed with Oligo dT primer using a HiScript^®^ II One Step RT-PCR Kit (Vazyme, China). qRT-PCR was performed on a Bio-Rad CFX96 in conjunction with SYBR Green I dye (Vazyme, China), while qPCR was performed on a Bio-Rad CFX96 Real-Time PCR Detection System (Bio-Rad, Hercules, CA). Primers were designed using Primer-BLAST (http://www.ncbi.nlm.nih.gov/tools/primer-blast/). The results were normalized to those of a housekeeping gene, *CsGAPDH*, and calculated by the comparative Ct method^36^. The primers used for real-time quantitative RT-PCR are listed in Supplementary Table S1.

### *CsLHT* expression in yeast cells

To isolate *CsLHT* genes, PCR was performed using cDNA derived from the roots of the tea plant cultivar Shuchazao (*Camellia sinensis* cv. Shuchazao). Primers were designed based on the *CsLHT* nucleotide sequences deposited in the tea plant genome database. For yeast complementation and uptake studies, *CsLHT* genes were cloned into a yeast pYES2 expression vector (Invitrogen, Carlsbad, CA) and confirmed by sequencing. The primers used for cloning are listed in Supplementary Table S1.

The method of plant gene transformation into yeast cells was as previously described by Hirner et al.^37^ and Dong et al.^38^. For amino-acid uptake studies, the *Saccharomyces cerevisiae* mutant strain 22Δ10α (*MATα gap1-1 put4-1 uga4-1 can1::HisG lyp1/alp1::HisG hip1::HisG dip5::HisG gnp1Δ agp1Δ ura3-1*) and wild-type strain 23344c were employed. Recombinant plasmids were transferred into these 22Δ10α mutants using a yeast transformation kit (Zymo Research, USA). Synthetic dropout (SD/-Ura) media were used, with 2% glucose as the carbon source for selection and normal growth and with 2% galactose for inducing the expression of *CsLHT* genes. Yeast cells were grown on nitrogen (N)-free media supplemented with ammonium sulfate or the respective test amino acid as the N source.

### Yeast amino-acid transport assays

Yeast amino-acid transport assays were performed, as previously described by Hirner et al.^37^ and Dong et al.^38^, with some modifications. For amino-acid uptake experiments, yeast cells were inoculated into YNB media consisting of 2 mM (NH_4_)_2_SO_4_ and grown to an OD_600_ of 1.0. An aliquot of these cells was then used to inoculate 50 mL of fresh media, after which the cells were allowed to grow overnight. The cells were then harvested at the desired optical density, washed three times with N-free growth media, and collected in the same media. The method for ^15^N isotope detection, using a DeltaV IRMS (Thermo Fisher Scientific, USA), was as previously described^39^.

Amino-acid uptake assays were initiated by adding yeast cells (4 mL aliquots) into tubes containing 200 µM ^15^N-labeled Glu. The yeast cells were collected at 30°C after 0, 2, 5, 10, 20, and 30 min of incubation. Kinetic analyses of amino-acid uptake were initiated by adding 4 mL mixtures of yeast cells into tubes containing 10, 20, 50, 100, 200, or 500 µM ^15^N-labeled Glu for *CsLHT1*-expressing cells and 0.5, 1, 2, 5, 10, or 20 mM ^15^N-labeled Glu for *CsLHT6*-expressing cells. These yeast cells were collected at 30°C after 10 min of incubation. Amino-acid uptake under different pH levels was initiated by adding 4 mL mixtures of yeast cells into tubes containing 200 µM ^15^N-Glu in media at a pH of 4, 5, 6, 7, or 8. Inhibitor experiments were performed under the same conditions, except that the incubation medium also included 0.1 mM carbonyl cyanide-m-chlorophenyl hydrazone (CCCP) or 0.1 mM diethylstilbestrol (DES). However, for competition experiments, the medium also included 2 mM unlabeled competitive amino acids. For these assays, yeast cells were collected at 30°C after 10 min of incubation.

### Subcellular localization of CsLHT1 and CsLHT6

Subcellular localization of amino-acid transporters was performed, as described previously by Santiago et al.^40^ and Li et al.^41^, with some modifications. To investigate subcellular localization, the coding sequences of *CsLHT1* and *CsLHT6* were amplified and cloned into a PK7WGFS2.0-GFP vector for transient expression in *Nicotiana benthamiana* leaves according to a previous report^35^. Three days after infiltration, GFP fluorescence was examined via an Olympus FV1000 confocal laser scanning microscope (Olympus Corporation, Beijing, China) using excitation and emission wavelengths of 484 nm and 507 nm, respectively.

### Overexpression of *CsLHT1* and *CsLHT6* in *Arabidopsis*

The coding DNA sequences of *CsLHT1* and *CsLHT6* were cloned into a pB2WG7 vector downstream of the Cauliflower mosaic virus *35* *S* promoter and then introduced into *Agrobacterium tumefaciens* (GV3101). Gene transformation into *Arabidopsis* was performed, as previously described by Zhang et al.^42^. The floral-dip method was used to generate *CsLHT1-* and *CsLHT6-*overexpressing *Arabidopsis* lines, as well as control plants carrying a pB2WG7,0 empty vector.

## Results

### Amino-acid composition in tea plantation soils

**Fig. 1:**
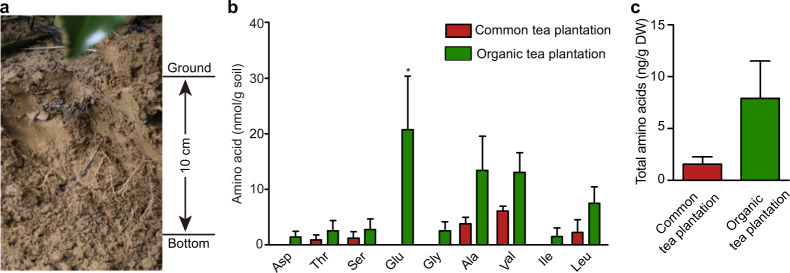
Amino-acid contents in soils of a conventional (control) tea plantation and an organic tea plantation. **a** Schematic diagram showing the upper region of the soil from which samples were collected in the present study. Soil samples up to 10 cm beneath the surface were collected. **b** Contents of various amino acids in soils collected from a conventional (control) and an organic tea plantation. **c** Total amino-acid contents in the soils of the two tea plantations. The data shown are the means ± SDs (*n* = three replicates)

Amino acids have been detected in many soil types^11–13^. However, there is little information on the amino-acid contents in tea plantation soils. In this study, soil samples were collected from control (common) tea plantation and an organic tea plantation to assess their amino-acid contents (Fig. 1a). Amino acids, including glutamate (Glu), alanine (Ala), valine (Val), leucine (Leu), threonine (Thr), serine (Ser), aspartate (Asp), glycine (Gly), and isoleucine (Ile), were detected in these soils (Fig. 1b). The levels of Glu, Ala, Val, and Leu were higher than those of the other amino acids. The total amino-acid content in the soils was 1–8 μg/g dry soil, with the amino-acid contents in the organic tea plantation soil being higher than those in the conventional tea plantation soil (Fig. 1b, c). These results confirmed the presence of amino acids in the soils of tea plantations, which is consistent with findings in other ecosystems, and implied a more important role of soil amino acids inorganic tea plantations.

### Glu feeding increases amino-acid levels in tea plant roots

**Fig. 2:**
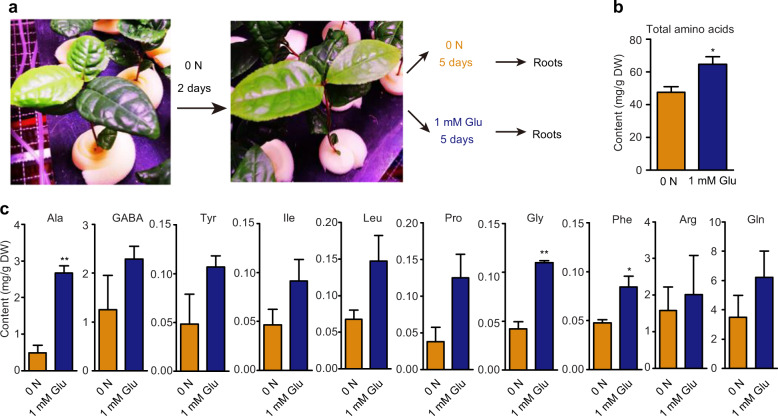
Amino-acid contents in the roots of tea seedlings fed exogenous Glu or subjected to N stress. **a** Schematic diagram of amino-acid feeding of hydroponically grown tea seedlings. Tea seedlings were first cultured under nitrogen deficiency (0 N) for 2 d, and then fed with 1 mM Glu for 5 d, with seedings kept under 0 N as the control. **b** Total amino-acid contents in the roots of tea seedlings under 0 N or fed 1 mM Glu. **c** Levels of various amino acids in the roots of tea seedlings under 0 N or fed 1 mM Glu. The data shown are the means ± SDs (*n* = 3). The asterisks represent statistical significance determined by Student’s *t* test (*p < 0.05; **p < 0.01)

Plant roots can take up various amino acids, such as Asp, Glu, and Gln, over a wide range of concentrations from 0.01 μM to 5 mM^37,43^. Exogenous Glu can support seedling growth and increase the contents of Glu, Gln, Asp, Ala, Ser, Asn, and γ-aminobutyric acid (GABA) in rice roots^44,45^. As shown in Fig. 1b, Glu was the most abundant amino acid in the soil of the organic tea plantations investigated in our study (Fig. 1b). We speculated that Glu is an important amino acid that can be directly utilized by tea roots. Therefore, we fed tea plants with Glu, as the sole N source, to test whether tea plants could use amino acids from the media. In this experiment, hydroponically cultured tea plant seedlings were first subjected to an N deficiency (0 N) for 2 d and then provided 1 mM Glu for 5 d, with 0 N for 5 d serving as the control (Fig. 2a). The roots of these seedlings were collected for amino-acid analyses, and we found that 1 mM Glu feeding increased the total amino-acid contents in these roots, especially Ala, GABA, tyrosine (Tyr), Ile, Leu, proline (Pro), Gly, phenylalanine (Phe), and Gln (Fig. 2b, c). These findings support the hypothesis that tea plants can acquire Glu from media as an N source and metabolize it to increase the synthesis of other amino acids.

### Absorption of ^15^N-labeled Glu by tea roots and identification of *CsLHT* genes

**Fig. 3:**
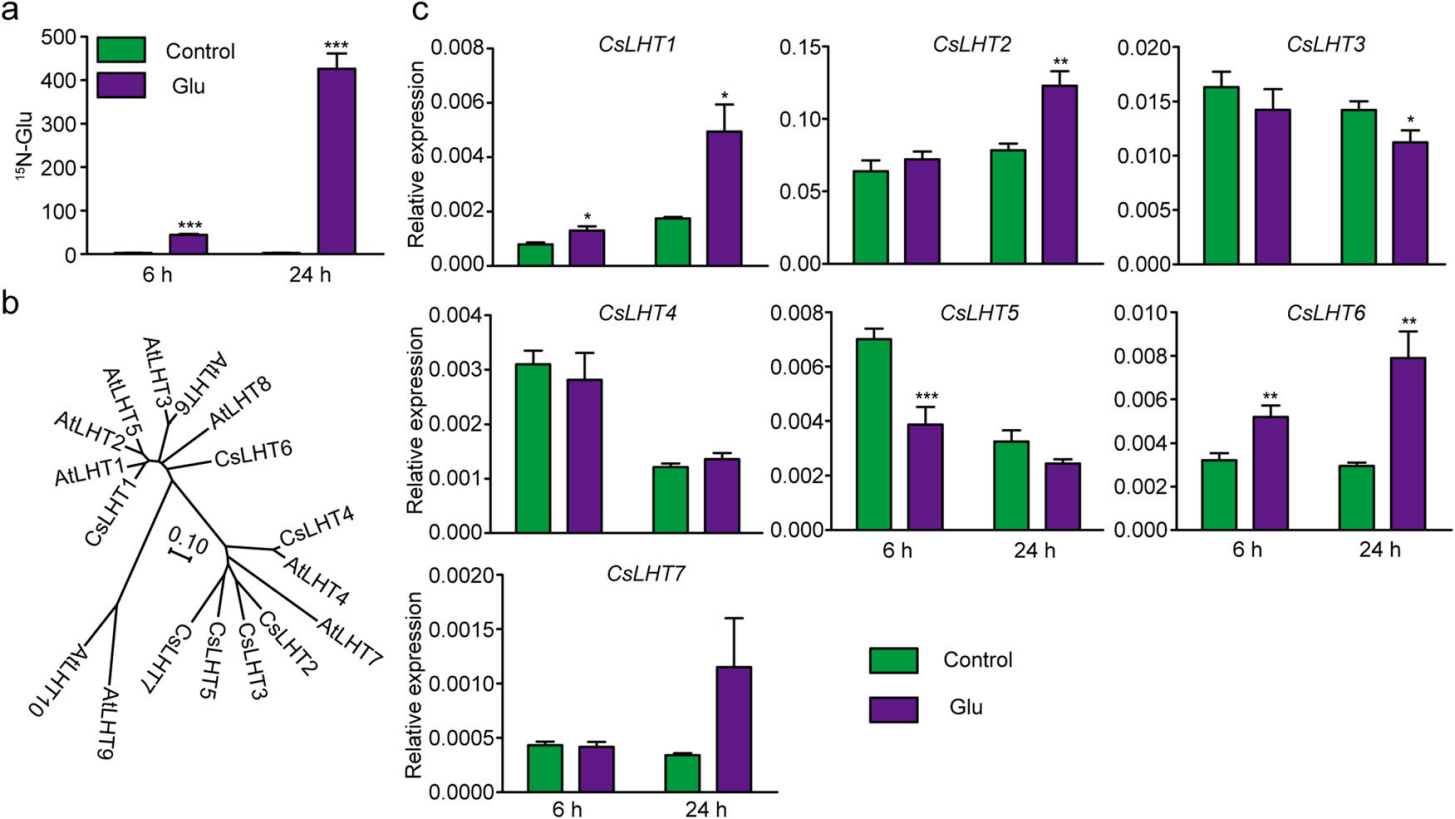
Uptake of ^15^N-Glu by tea seedling roots and the response of *CsLHT* genes in the roots to Glu feeding. **a** ^15^N-Glu content in the roots of tea seedlings fed exogenously with 2 mM ^15^N-Glu for 6 h or 24 h or in the roots of seedlings under 0 N (control). **b** Phylogenetic relationships among CsLHT and AtLHT genes. The genes and their sequence IDs are as follows: AtLHT1 (AT5G40780), AtLHT2 (AT1G24400), AtLHT3 (AT1G61270), AtLHT4 (AT1G47670), AtLHT5 (AT1G67640), AtLHT6 (AT3G01760), AtLHT7 (AT4G35180), AtLHT8 (AT1G71680), AtLHT9 (AT1G25530), AtLHT10 (AT1G48640), CsLHT1 (TEA026462), CsLHT2 (TEA021847), CsLHT3 (TEA033469), CsLHT4 (TEA029168), CsLHT5 (TEA016092), CsLHT6 (TEA003706), and CsLHT7 (TEA021821). Multiple sequence alignment of full-length proteins was performed, and the phylogenetic tree was constructed using MEGA 6 software. The scale bar represents a 10% amino-acid substitution rate. **c** Relative expression of *CsLHT* genes in the roots of tea seedlings fed exogenously with 2 mM ^15^N-Glu for 6 h or 24 h or in the roots of seedlings under 0 N (control). *CsGAPDH* was used as an internal control. The data represent the means ± SDs (*n* = 3). The asterisks represent statistical significance determined by Student’s *t* test (*p < 0.05; **p < 0.01; ***p < 0.001)

To test whether tea plant roots can acquire amino acids from the soil, we fed hydroponically cultured tea plant seedlings ^15^N-labeled Glu by adding ^15^N-Glu to the nutrient solution. After 6 h or 24 h, the ^15^N levels in the roots increased significantly compared with those in the control roots (Fig. 3a). These results indicated that tea seedlings can directly take up exogenously applied Glu.

As AtLHT1 and AtLHT6 can mediate amino-acid uptake from the soil^25,38^, we hypothesized that CsLHTs are involved in amino-acid uptake from the soil in the tea plant. To test this hypothesis, we first identified LHT family members in the tea plant. To this end, we searched for “lysine histidine transporter” in the gene annotations of transcriptome data for tea plant roots^38^ and identified 13 putative CsLHTs. We next downloaded AtLHT sequence information from The Arabidopsis Information Resource website (www.arabidopsis.org) and used it to identify CsLHT homologs via an online BLAST search^39^ (http://tpia.teaplant.org/). In this way, we identified two additional CsLHTs. We successfully cloned seven of these 15 *CsLHT* genes, and they were named *CsLHT1*-*CsLHT7* based on their homologies to *AtLHT*s (Fig. 3b).

### Expression of *CsLHT1*, *CsLHT2,* and *CsLHT6* in the roots was induced by Glu feeding

To investigate whether the expression of *CsLHTs* in the roots responds to amino acids in the soil, 100-day-old tea seedlings were transferred to N-deficient media supplemented with 2 mM Glu for 6 h or 24 h. Our results revealed that root expression of *CsLHT1* and *CsLHT6* was induced at both time points upon Glu feeding (Fig. 3c). Root expression of *CsLHT2* was induced at 24 h, whereas *CsLHT3*, *CsLHT4*, *CsLHT5*, and *CsLHT7* did not respond to or were repressed by Glu feeding. Hence, these *CsLHT* genes respond differentially to amino acids within the root media, which may reflect various roles for these genes in tea root physiology. Based on these findings, we proposed that CsLHT1, CsLHT2, and CsLHT6 likely function in the root uptake of amino acids from the soil.

### CsLHT1 and CsLHT6 have the capacity to transport amino acids

**Fig. 4:**
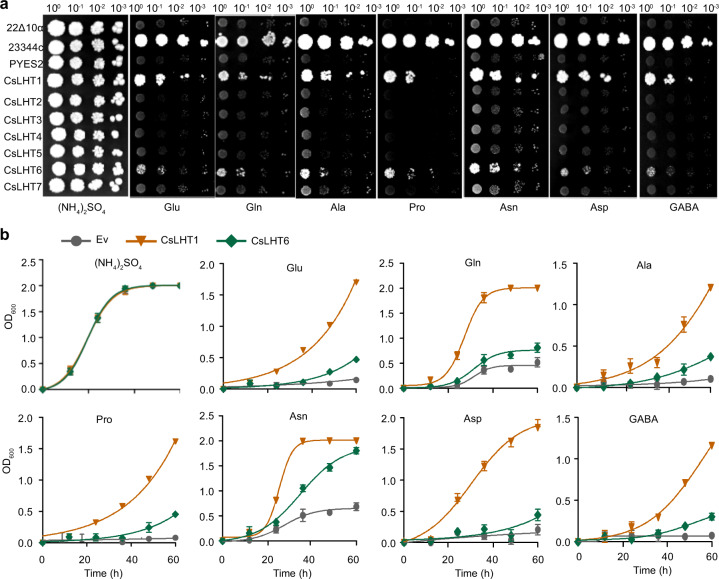
CsLHT1 and CsLHT6 enable the growth of yeast strain 22Δ10α on media with amino acids constituting the sole N source. Amino-acid transport-defective yeast strain 22Δ10α (genotype MATα *gap1-1 put4-1 uga4-1 can1::HisG lyp1-alp1::HisG hip1::HisG dip5::HisG gnp1Δ agp1Δ ura3-1*) was employed to analyze the growth of yeast cells expressing *CsLHT* genes and supplemented with the indicated amino acids (at 2 mM) as the sole N source. 23344 C was used as the wild-type yeast strain. Ammonium sulfate, at 2 mM, served as the control N source. pYES2 empty vectors were used. The *CsLHT* genes were cloned into pYES2 and then transferred into 22Δ10α. **a** Yeast growth on YNB solid media. **b** Yeast growth on YNB liquid media

To identify the CsLHT(s) responsible for amino-acid uptake, we next tested whether these CsLHTs could transport amino acids. The yeast mutant strain 22Δ10α lacks 10 amino-acid transporter genes and cannot grow in media with amino acids (except arginine) constituting the sole N source^46^. Thus, we transferred the *CsLHT* genes individually into 22Δ10α and tested whether each transformant could grow in media with Glu, Gln, Ala, Pro, Asn, Asp or GABA as the sole N source. Interestingly, the expression of only *CsLHT1* or *CsLHT6* in this mutant enabled growth, indicating that CsLHT1 and CsLHT6 can transport these amino acids into yeast cells (Fig. 4a). Furthermore, according to these assays, yeast cells expressing *CsLHT1* exhibited enhanced growth relative to that of cells expressing *CsLHT6* (Fig. 4a). Similar findings were obtained from liquid culture assays (Fig. 4b). These results support the notion that CsLHT1 has a higher affinity for amino acids than does CsLHT6.

### CsLHT1 and CsLHT6 have different affinities for amino acids

**Fig. 5:**
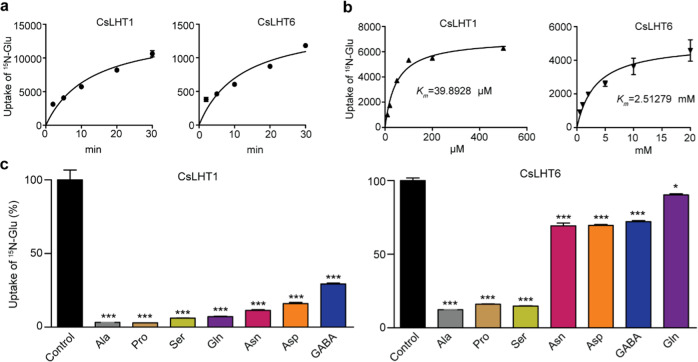
Kinetic and substrate specificity analyses of amino-acid transport mediated by CsLHT1 and CsLHT6. **a** Time course of CsLHT1- and CsLHT6-mediated uptake of ^15^N-Glu into 22Δ10α mutant cells. **b** Determination of *K*_*m*_ values of CsLHT1/6 for ^15^N-Glu in yeast. The data represent the means ± SDs (*n* = 3). **c** Substrate specificity of CsLHT1 and CsLHT6. ^15^N-Glu uptake was measured in the presence of a 10-fold excess of the indicated competitors. ^15^N-Glu uptake without competitive amino acids was set to 100%. The data represent the means ± SDs (*n* = 3). The asterisks represent statistical significance determined by Student’s *t* test (*p < 0.05; ***p < 0.001)

To assess the CsLHT1 and CsLHT6 affinities for amino acids, we next analyzed their transport kinetics for ^15^N-Glu. Here, the yeast mutant 22Δ10α, harboring CsLHT1 or CsLHT6, was fed 200 μM ^15^N-Glu, and the ^15^N taken up into these cells was measured after a 0, 2, 5, 10, 20, or 30 min incubation period (Fig. 5a). As expected, these transport assays demonstrated that ^15^N-Glu was absorbed and accumulated in the cells expressing *CsLHT1* or *CsLHT6*. Importantly, ^15^N-Glu accumulation increased in the cells in a time-dependent manner. Moreover, ^15^N-Glu accumulation in cells expressing *CsLHT1* was much higher than that in cells expressing *CsLHT6*. These results provided further support for the notion that CsLHT1 and CsLHT6 can transport amino acids, albeit with different affinities.

A ^15^N-Glu concentration series (10, 20, 50, 100, 200, and 500 µM) for CsLHT1 and one (0.5, 1, 2, 5, 10, and 20 mM) for CsLHT6 was also employed in our transport kinetics assays; ^15^N levels were measured in the cells after a 10 min incubation. Concentration-dependent accumulation of ^15^N-Glu in the cells was observed (Fig. 5b). The kinetic parameters calculated based on these ^15^N-Glu assays indicated *K*_*m*_ values for CsLHT1 and CsLHT6 of 39.9 μM and 2.5 mM, respectively. These data indicated that CsLHT1 acts as a high-affinity-amino-acid transporter, whereas CsLHT6 is a low-affinity transporter.

To analyze CsLHT1 and CsLHT6 substrate specificities, competition experiments were next performed in which ^15^N-Glu uptake was measured in the presence of a 10-fold excess of unlabeled competitive amino acids. These assays showed that unlabeled Ala, Pro, Ser, Gln, Asn, Asp, and GABA significantly repressed ^15^N-Glu uptake by CsLHT1. In contrast, only Ala, Pro, and Ser significantly repressed ^15^N-Glu uptake by CsLHT6 (Fig. 5c). These findings indicated that, as a high-affinity-amino-acid transporter, CsLHT1 has broader substrate specificity than does the low-affinity-amino-acid transporter CsLHT6.

### CsLHT1 and CsLHT6 are H^+^-coupled amino-acid transporters

**Fig. 6:**
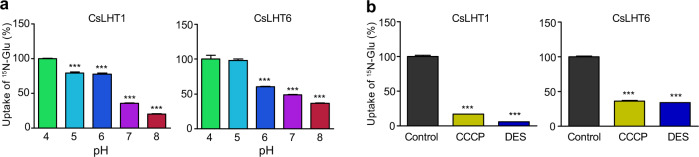
pH dependence of ^15^N-Glu uptake into 22Δ10α yeast mutants expressing *CsLHT1* or *CsLHT6*. **a** ^15^N-Glu uptake by CsLHT1 and CsLHT6 under various pH conditions. The uptake at a pH of 4 was set as 100%. The data represent the means ± SDs (*n* = 3). **b** ^15^N-Glu uptake by CsLHT1 and CsLHT6 in the presence of an H^+^-ATPase inhibitor, diethylstilbestrol (DES), and a protonophore, carbonyl cyanide-m-chlorophenyl hydrazone (CCCP). Yeast cells were incubated for 10 min in YNB media + ^15^N-Glu, with or without DES or CCCP. ^15^N-Glu uptake without DES or CCCP was used as a control. The data represent the means ± SDs (*n* = 3). The asterisks represent statistical significance determined by Student’s *t* test (***p < 0.001)

To explore the mechanism through which CsLHT1 and CsLHT6 transport amino acids, we next performed ^15^N-Glu uptake assays at pH levels of 4.0, 5.0, 6.0, 7.0, and 8.0. These assays revealed that CsLHT1 and CsLHT6 ^15^N-Glu transport activity was high at low pH levels but was significantly lower at neutral and alkaline pH levels (Fig. 6a). Moreover, the addition of the H^+^-ATPase inhibitor DES and the protonophore CCCP nearly abolished ^15^N-Glu uptake by CsLHT1 and CsLHT6 (Fig. 6b). These findings support the hypothesis that CsLHT1 and CsLHT6 function as amino acid-proton cotransport systems.

### CsLHT1 and CsLHT6 are highly expressed in the roots and are localized to the plasma membrane

**Fig. 7:**
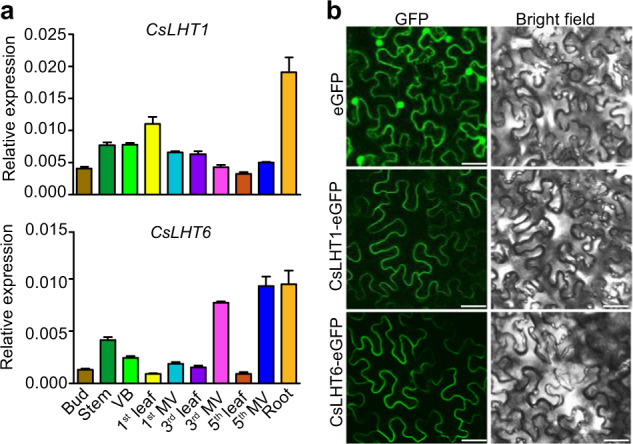
Tissue-specific expression of *CsLHT1* and *CsLHT6* and their subcellular localization. **a** Expression of *CsLHT1/6* in different tea plant tissues. *MV* major vein, *VB* vascular bundle, extracted from the indicated stem. The data represent the means ± SDs (*n* = 3). **b** CsLHT1-eGFP fusion protein, CsLHT6-eGFP fusion protein and eGFP control transiently expressed in tobacco (*N. benthamiana*) leaves. Bars = 25 µm

Tissue-specific expression of genes is generally linked to the biological functions of those genes. For example, *AtLHT1* and *AtLHT6*, functioning in the uptake of amino acids from the soil in *Arabidopsis*, are highly expressed in roots^25,37,47^. To assess this relationship for *CsLHT1* and *CsLHT6*, we evaluated their expression in many tea plant tissues, including the leaf buds; first, third and fifth leaves and major veins (MV) (1st leaf and 1st MV; 3rd leaf and 3rd MV; 5th leaf and 5th MV); stems; and vascular bundles extracted from both stem and root tissues (Fig. 7a). Here, *CsLHT1* and *CsLHT6* were most highly and primarily expressed in the roots, supporting the notion that CsLHT1 and CsLHT6 act in the uptake of amino acids from the soil.

To further explore the mechanism of amino-acid uptake by CsLHT1 and CsLHT6, we examined the subcellular localization of CsLHT1 and CsLHT6. Here, the CaMV *35* *S* promoter-driven enhanced green fluorescent protein (eGFP)-CsLHT1 or eGFP-CsLHT6 fusion protein and CaMV *35* *S* promoter-driven eGFP were transiently expressed in epidermal cells of tobacco leaves. Florescent signals of CsLHT1-eGFP and CsLHT6-eGFP were observed at the plasma membrane (Fig. 7b). In contrast, free eGFP florescent signal was distributed within both the cytoplasm and the nucleus. Based on these cellular assays, it appears that CsLHT1 and CsLHT6 are plasma membrane-localized amino-acid transporters, the conclusion of which is also supported by our finding that CsLHT1 and CsLHT6 import amino acids into root cells.

### Overexpression of *CsLHT1* and *CsLHT6* in *Arabidopsis* increases amino-acid uptake

**Fig. 8:**
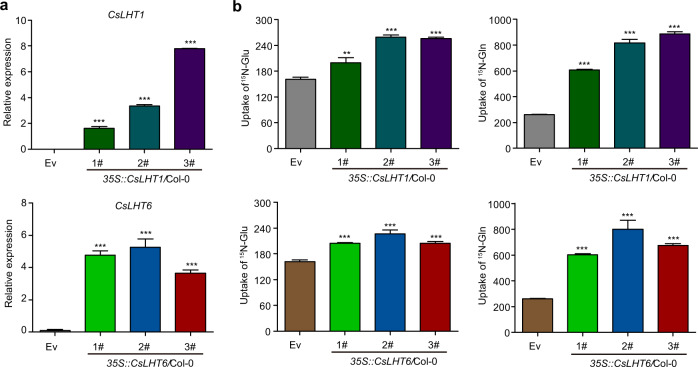
^15^N-Glu and ^15^N-Gln uptake by *Arabidopsis* lines overexpressing *CsLHT1* and *CsLHT6*. **a** Expression levels of *CsLHT1* and *CsLHT6* in *35* *S::CsLHT1* and *35* *S::CsLHT6* transgenic *Arabidopsis* lines. *Arabidopsis* transformed with an empty vector (Ev) served as the negative control. The data represent the means ± SDs (*n* = 3). **b** Uptake of ^15^N-Glu and ^15^N-Gln in Ev-, *CsLHT1-* and *CsLHT6-*overexpressing *Arabidopsis* lines. The data represent the means ± SDs (*n* = 3). The asterisks represent statistical significance determined by Student’s *t* test (***p < 0.001). The numbers represent individual transgenic lines

Currently, gene transformation into tea plants remains impractical. Hence, to further verify that CsLHT1 and CsLHT6 participate in amino-acid uptake from the soil, *CsLHT1* and *CsLHT6* were overexpressed in the model plant species *Arabidopsis* ecotype Columbia-0 (Col-0) (Fig. 8a). Compared with the empty vector-expressing line, three *CsLHT1-* or *CsLHT6*-overexpressing lines presented improved ^15^N-Glu or ^15^N-Gln uptake capacity (Fig. 8b). Thus, overexpression of *CsLHT1* and *CsLHT6* improved amino-acid uptake in planta. Taken together, our findings provide support for a model in which CsLHT1 and CsLHT6 play important roles in amino-acid uptake from the soil into the roots of the tea plant.

## Discussion

N is one of the most important mineral macronutrients essential for plant growth and development. In this regard, it is well known that plants have evolved the capacity to absorb N in the form of amino acid, from aqueous solutions^12,48^. Furthermore, root uptake of amino acids is known to be energy-dependent and regulated by the concentration of amino acids in solution, indicating that their uptake is an active process mediated by specific transporters^16–18,37^. In this study, we determined that higher amino-acid levels are present in the soil of an organic tea plantation compared with a conventional tea plantation. Furthermore, ^15^N-Glu-feeding experiments indicated that tea plants can absorb exogenously applied amino acids that can then be used for N assimilation. In addition, we demonstrated that CsLHT1 and CsLHT6 are involved in the uptake of amino acids from the soil in the tea plant.

### Amino acids constitute an important N pool inorganic tea plantations

It has been suggested that tea plants grown inorganic tea plantations are subjected to N-deficient conditions due to the absence of inorganic fertilizer^9^. Compared with conventional tea, that produced under organic management systems contains higher levels of catechins that are linked to antioxidant effects of tea infusions. However, organic tea contains lower levels of amino acids that are also important compounds in terms of tea quality^9^. The decay of large amounts of pruned tea shoots may contribute significantly to soil amino-acid levels inorganic tea plantations; the decomposition of such organic matter and nutrient recycling depends largely on soil fungi^43,44^. Interestingly, the long-term application of high amounts of N fertilizer was found to reduce soil fungal diversity in tea plantations^49,50^. This likely could account for why we observed higher amino-acid contents in the organic tea plantation compared with the conventional tea plantation (Fig. 1). This implies a more important role for soil amino acids in tea plant grown inorganic tea plantations.

### Soil amino acids can be used as sources of N for the tea plant

It has been reported that, in addition to inorganic N, amino acids can support tree growth^51,52^. As a perennial evergreen tree species, the tea plant can also use organic fertilizer. However, the role of soil amino acids in tea plant growth and metabolism has not yet been investigated. In this study, we observed that the tea plant could take up ^15^N-Glu, and Glu feeding increased the amino-acid contents in the roots (Figs. 2 and 3a). This revealed that tea plants can take up amino acids from the soil for use in the synthesis of other amino acids. In our study, nine amino acids were detected in the soil of an organic tea plantation, and the utilization of exogenous Glu was analyzed in detail. In future studies, it will be important to test the roles of various mixtures of amino acids for use as fertilizers for the growth and metabolism of the tea plant.

### Amino acid-induced CsLHT1 and CsLHT6 take up amino acids from the soil in the tea plant

Amino-acid transport in plants involves a number of processes, including uptake from the soil solution, cell-to-cell transport across the root cortex and into the stele, xylem loading, root-to-shoot delivery through the transpiration stream, xylem-to-phloem transfer, translocation via the phloem loading to sink regions of the plant, and phloem unloading, and post-vascular movement into sink cells^53^. These processes are mediated by plasma membrane-localized amino-acid transporters. In plants, there are hundreds of amino-acid transporters belonging to many families. Among these families, the AAPs, LHTs, cation amino-acid transports (CATs), Proline Transporters (ProTs), and Usually Multiple Acids Move In and out Transporters (UMAMITs) have been functionally well characterized^54^. In *Arabidopsis*, LHT1, LHT6, AAP1, AAP5, ProT2 mediate amino-acid uptake from the soil; AAP2 and AAP6 act in amino acid xylem and phloem transfer; AAP2, AAP3, AAP5, AAP8, ProT1, CAT1, CAT6, CAT9 are involved in amino acid phloem loading; UmamiT14, UmamiT18, UmamiT28 and UmamiT29 functions in amino acid phloem export into the embryo^54^.

The molecular mechanism underlying the uptake of amino acids from the soil by trees has not been thoroughly studied. In this study, we identified seven CsLHTs that were grouped into two clusters, which was consistent with LHTs in *Arabidopsis* (Fig. 3b). CsLHT1 and CsLTH6 in cluster I have amino-acid transport activity (Fig. 5), which is also consistent with AtLHT1 and AtLHT6. Moreover, these two genes were highly expressed in the roots and both encode plasma membrane-localized proteins (Fig. 7). These findings support the hypothesis that CsLHT1 and CsLHT6 play important roles in amino-acid uptake from the soil (Figs. 4–8). However, the members of cluster II, CsLHT2, CsLHT3, CsLHT4, CsLHT5, and CsLHT7, did not display amino-acid transport activity (Fig. 5). Interestingly, except for AtLHT1 and AtLHT6, there are no other AtLHTs being shown to transport amino acids. It is possible that cluster II LHTs are involved in the transport of metabolites other than amino acids. For example, AtLHT2 was recently shown to transport 1-aminocyclopropane-1- carboxylic acid, a biosynthetic precursor of ethylene, in *Arabidopsis*^55^.

### CsLHT1 and CsLHT6 may play different roles in the uptake of amino acids from the soil

LHT1 has been thoroughly characterized as a high-affinity-amino-acid transporter and has a major role in the uptake of amino acids from the soil in both *Arabidopsis* and rice^27,29,33,37,43,47^. In contrast, there is only one report on the function of *AtLHT6*^25^; it is highly expressed in the roots, and the *atlht6* mutant presented reduced amino-acid uptake from media when supplied with a high amount of amino acids. Although the authors did not characterize the amino-acid transport kinetics for AtLHT6, their results are consistent with this protein being a low-affinity-amino-acid transporter.

In the present study, we characterized CsLHT1 to be a high-affinity amino-acid transporter (*K*_*m*_ ~40 μM for ^15^N-Glu), with a capacity to transport a broad spectrum of amino acids (Figs. 4 and 5). By contrast, CsLHT6 exhibited a much lower affinity (*K*_*m*_ ~2.5 mM) for ^15^N-Glu, and it also displayed higher substrate specificity. Considering that amino-acid concentrations in the soil of tea plantations are low (<30 μM) (Fig. 1), CsLHT1 may play a more important function than CsLHT6 in the uptake of amino acids from the soil into tea plants. However, in soils, amino-acid contents could be much higher, locally, particularly in the vicinity of decomposing animal or vegetable matter^56^. In this situation, CsLHT6 may play an important role in the uptake of amino acids. In addition, *CsLHT6* is also highly expressed in the major veins of mature leaves (Fig. 7a), suggesting a role for CsLHT6 in amino-acid transport within these tea leaves.

Given that protocols for the efficient production of transgenic tea cultivars are lacking, *CsLHT1* and *CsLHT6* expression cannot be modulated by either overexpression or CRISPR/Cas9 gene editing. However, in China, there is an abundance of tea plant germplasm resources. *CsLHT1* and *CsLHT6* are potential gene markers for selecting germplasms that can efficiently take up amino acids. Moreover, germplasms with high *CsLHT1* or *CsLHT6* expression can be used as rootstocks for grafting with elite cultivars to improve the ability of these cultivars to take up amino acids from the soil. Alternatively, these germplasms can be utilized through gene introgression. These grafted lines that can efficiently take up amino acids or novel cultivars should be better suited for use inorganic tea plantations than in conventional tea plantations.

## Supplementary information

Primers used in this study
