# Supplementary material for: The tea plant CsLHT1 and CsLHT6 transporters take up amino acids, as a nitrogen source, from the soil of organic tea plantations
Source: Hortic Res. 2021 Aug 1;8:178. doi: 10.1038/s41438-021-00615-x (PMC8325676; doi:10.1038/s41438-021-00615-x)
Supplement: Supplementary file 1 — Primers used in this study [file 41438_2021_615_MOESM1_ESM.doc]

**SUPPORTING INFORMATION**

**Table S1. Primers used for yeast expression vector construction**

| gene ID | Primers sequences |
| --- | --- |
| CsLHT1 | F 5’-CCCAAGCTTATGGCACCTCAAGCTCCA -3’  R 5’-CCGCTCGAGCTAAGAGTAGAATTGATAGCCCT -3’ |
| CsLHT2 | F 5’- CCCAAGCTTATGGGTGAGGTTGACGAGG -3’  R 5’- CCGCTCGAGTTACTGAGGCTTAAAGAAGCTGA -3’ |
| CsLHT3 | F 5’- GGAATTCATGGAGGAGAGGCCGGAA -3’  R 5’- GCTCTAGATTAATTGGGCTTAAAGAATTTGAG -3’ |
| CsLHT4 | F 5’- GGAATTCATGAGTGAAGTGCAGGAGATA -3’  R 5’- CCCTCGAGCTAGACAGCAGGCTTGAAGAA -3’ |
| CsLHT5 | F 5’- GGAATTCATGGATGTGGTGGTAGAAGTG -3’  R 5’- CCCTCGAGCTAGGGTTTGAAGAAGTGCAC -3’ |
| CsLHT6 | F 5’- CCCAAGCTTATGGTTTCAAACTCTCCACCAG -3’  R 5’- GGAATTCTCATGAGTAGAACTCATAGGTAGAGGA -3’ |
| CsLHT7 | F 5’- CCCAAGCTTATGGGAGAAGTGGGTGAAGTA-3’  R 5’- CCCTCGAGTTAGTGAGGCCTAAAGAAATT -3’ |

**Table S2.** Primers for overexpression and subcellular localization vector construction

| gene ID | Primers sequences |
| --- | --- |
| CsLHT1 | F 5’-GGGGACAAGTTTGTACAAAAAAGCAGGCTTCATGGCACCTCAAGCTCCA-3’  R 5’-GGGGACCACTTTGTACAAGAAAGCTGGGTCCTAAGAGTAGAATTGATAGCCCT-3’ |
| CsLHT6 | F 5’-GGGGACAAGTTTGTACAAAAAAGCAGGCTTCATGGTTTCAAACTCTCCACCAG-3’  R 5’-GGGGACCACTTTGTACAAGAAAGCTGGGTCTCATGAGTAGAACTCATAGGTAGAGGA-3’ |

**Table S3. Primers for qRT-PCR**

| gene ID | Primers sequences |
| --- | --- |
| CsLHT1 | F 5’- TCTGAGAAGGAGAAAGCAATCG -3’  R 5’- CAGAAACACCAGGACCCCAT -3’ |
| CsLHT2 | F 5’- TTATCGGAGGGATCGCGTTG -3’ |
|  | R 5’- GCGATCACTACCCCACTGAG -3’ |
| CsLHT3 | F 5’- GCGTCGAAAGAAGCGAAGTC -3’ |
|  | R 5’- TTCGTGAGATGCCCAACCTC -3’ |
| CsLHT4 | F 5’- AGGATGAAGAAGCCGTGCTC -3’ |
|  | R 5’- TGCTAGGCTTCCCAAGAACG -3’ |
| CsLHT5 | F 5’- CAGCCTCAACTCTGGGATCG -3’ |
|  | R 5’- GTTGCCACACAAAAGCCACT -3’ |
| CsLHT6 | F 5’- GCTTGAGCAAAGGGCGAGTT -3’  R 5’- TTGAGGGCATTTCAGGGGT -3’ |
| CsLHT7 | F 5’- TGGGGTATGGTGTGCTTGTC -3’ |
|  | R 5’- AAAGGCTGCAATGGCTAGGT -3’ |
